# Severe Case of Spongy Iritis in a Horse

**Published:** 1883-01

**Authors:** Edward T. Ely

**Affiliations:** Physician to the Manhattan Eye and Ear Hospital, N. Y.; 20 East 30th Street, New York


### CASE DEPARTMENT.

### I.—A SEVERE CASE OF SPONGY IRITIS IN A HORSE.

REPORTED BY EDWARD T. ELY, M.D.,
*Physician to the Manhattan Eye and Ear Hospital, N. Y.*

Last winter I was asked by Dr. S., of this city, to examine his horse—a very valuable animal—which had suddenly become entirely blind. The history was that the horse had taken cold during the stormy weather prevailing at the time, and that, while suffering in this way, the affection of the eyes had appeared.

I found the eyes considerably congested and the anterior chamber of each completely filled with a yellowish, jelly-like exudation which prevented any view of the deeper parts. Vision was reduced to bare perception of light, the animal groping about in a pitiful way and trembling with fear when touched. The objective symptoms were exactly like those of the disease known as *spongy iritis* in man, and on the strength of this resemblance I ventured to predict that the sight would be recovered.

I advised the instillation of a two-grain solution of atropine every two hours and the frequent use of hot water fomentations. This treatment was faithfully applied by the groom day and night. The hot water was applied for fifteen to twenty minutes at a time by means of a sponge. Within two days the exudation began to disappear so that parts of the iris could be seen and perfect recovery speedily followed. The groom and the other employees at the stable where the horse boarded were constantly suggesting the application of "a little alum," and they considered the patient doomed because their suggestion was not adopted. Any astringent application would certainly have been very bad treatment.

The behaviour of the animal during his illness, his fright and awkardness while blind and his evident happiness as sight returned were very interesting to witness.

The case seems to me to be interesting enough to record, although similar cases may be common in the experience of veterinary surgeons.

20 East 30th Street, New York, Oct., 1882.

